# Challenges and Controversies in COVID-19: Masking the General Population may Attenuate This Pandemic's Outbreak

**DOI:** 10.3389/fpubh.2021.643991

**Published:** 2021-09-08

**Authors:** Björn Johansson

**Affiliations:** ^1^Theme Aging, Karolinska University Hospital, Stockholm, Sweden; ^2^Department of Clinical Neuroscience, Karolinska Institutet, Stockholm, Sweden

**Keywords:** mask, simulation–computers, visualization, health policies, innovation, COVID-19, side-effects, non-linear systems

## Abstract

SARS-CoV-2, the virus that causes COVID-19, spreads i. a., by respiratory droplets. The use of masks in preventing spread is controversial; masks are considered useless by many, while being mandated in some locations. Here, the effect of masking the general population on a COVID-19-like epidemic is estimated by computer simulation using three separate types of software. The main questions are whether mask use by the general population can limit the spread of SARS-CoV-2 in a country and how to identify opportunities when mask use is cost-effective and safe. To address these questions, the protective effects of different types of masks, the side-effects of masks, and avenues for improvements of masks and masking are addressed. Main results: (i) Any type of mask, even simple home-made ones, may be of value, even if the protective effect of each mask (here dubbed “one mask-protection”) is low. Strict adherence to mask use does not appear to be critical but increasing one mask-protection to >50% was found to be advantageous. (ii) Masks do seem to reduce the number of new cases even if introduced at a late stage in an epidemic, but early implementation helps reduce the cumulative and total number of cases. (iii) The simulations suggest that it might be possible to eliminate a COVID-19 outbreak by widespread mask use during a limited period. There is a brief discussion of why the reported effect size of masking varies widely, and is expected to do so, because of different filtration abilities of different masks, differences in compliance and fitting, other routes of transmission, pre-existing immunity, and because a system of interconnected, disease-prone individuals has non-linear properties. A software solution to visualize infection spread is presented. The results from these simulations are encouraging, but do not necessarily represent the real-life situation, so it is suggested that clinical trials of masks are now carried out while continuously monitoring effects and side-effects. As mask use is not without risks and costs, it is suggested that governments and scientists have an important role in advising the public about the sensible use of masks.

## Introduction

Early in the COVID-19 epidemic, the World Health Organization recommended that face masks should only be used by health workers and people with confirmed or suspected coronavirus infection and their carers (1). Certain news items then discouraged the use of face masks (2). However, the WHO quickly changed opinion, and in China, which was reportedly very successful in containing the COVID-19 epidemic, there was widespread use of face masks, including by asymptomatic people (3). Hand hygiene is deemed the cornerstone of infection prevention (4). However, hand hygiene was promoted even before it was definitely known which procedures were active specifically for SARS-CoV-2, as discussed (4). It was noted that definitive claims on the effectiveness of various disinfectants against SARS-CoV-2 could not be made, simply because this is a new virus and the range of disinfectants had never been tested for SARS-CoV-2. Like other respiratory viruses, the new coronavirus spreads from person to person through airborne droplets, but other routes are known or suspected such as surfaces, where it can survive for days (5), so that touching infected surfaces can spread the virus [e.g., (4)].

Simple experiments suggest that masks may be effective against respiratory infections. For example, Johnson and colleagues had participants cough five times onto a Petri dish containing viral transport medium [cited in (6)]. Influenza virus could be detected by RT–PCR from all nine volunteers without a mask; no influenza virus could be detected when participants wore surgical or N95 masks. The same review concluded that there is some evidence to support the wearing of masks or respirators during illness to protect others. Tissue from a surgical mask was found to reduce the risk of COVID-19 transmission in hamsters (7). Hui et al. (8) found that masks can reduce the distance traveled by expelled air during a cough. Tracht and colleagues noticed that people are willing to wear face masks to protect themselves against infection (9). Using mathematical modeling, they concluded that if N95 respirators are 20% effective in reducing susceptibility and infectivity and 10% of the population wear them, the number of H1N1 cases is reduced by 20%.

A variety of masks and related devices exist, designed to protect the wearer or the environment. Medical masks, unfitted and disposable, can be used by infected individuals, healthcare professionals, or laymen to lower the transfer of infectious agents (10). Surgical masks are intended to limit contamination of wounds in surgery. A respirator, a type of mask, is fitted, can be disposable or reusable and protects the wearer against inhalation of harmful material. The National Institute for Occupational Safety and Health (NIOSH) regulates testing and certification of masks and similar respiratory protection equipment (11). In the European Union, similar standards are provided by the European Committee for Standardization. The NIOSH tests requires a minimum filtration efficiency of 95, 99, or 99.97% for an aerosol test (see standards for detailed specifications). The more protective masks may offer noticeable resistance to breathing and related to this, some individuals may find them difficult to wear for extended periods. The N95 respirator is a common mask that nominally filters at least 95% of airborne particles (12). It is indeed possible to obtain very close to 100% protection from respiratory pathogens such as SARS-CoV-2 using a Self-Contained Breathing Apparatus (SCBA). These are costly and require special training (often used by firefighters), see (13). However, an advanced mask, not SCBA, was tested with standardized methods, with influenza, rhinovirus, bacteriophage, *Staphylococcus aureus*, and model pollutants (14), >99.7% efficiency was found for the exclusion of influenza A virus, rhinovirus 14, and *S*. *aureus*, and >99.3% efficiency for paraffin oil and sodium chloride.

**Figure 1:**
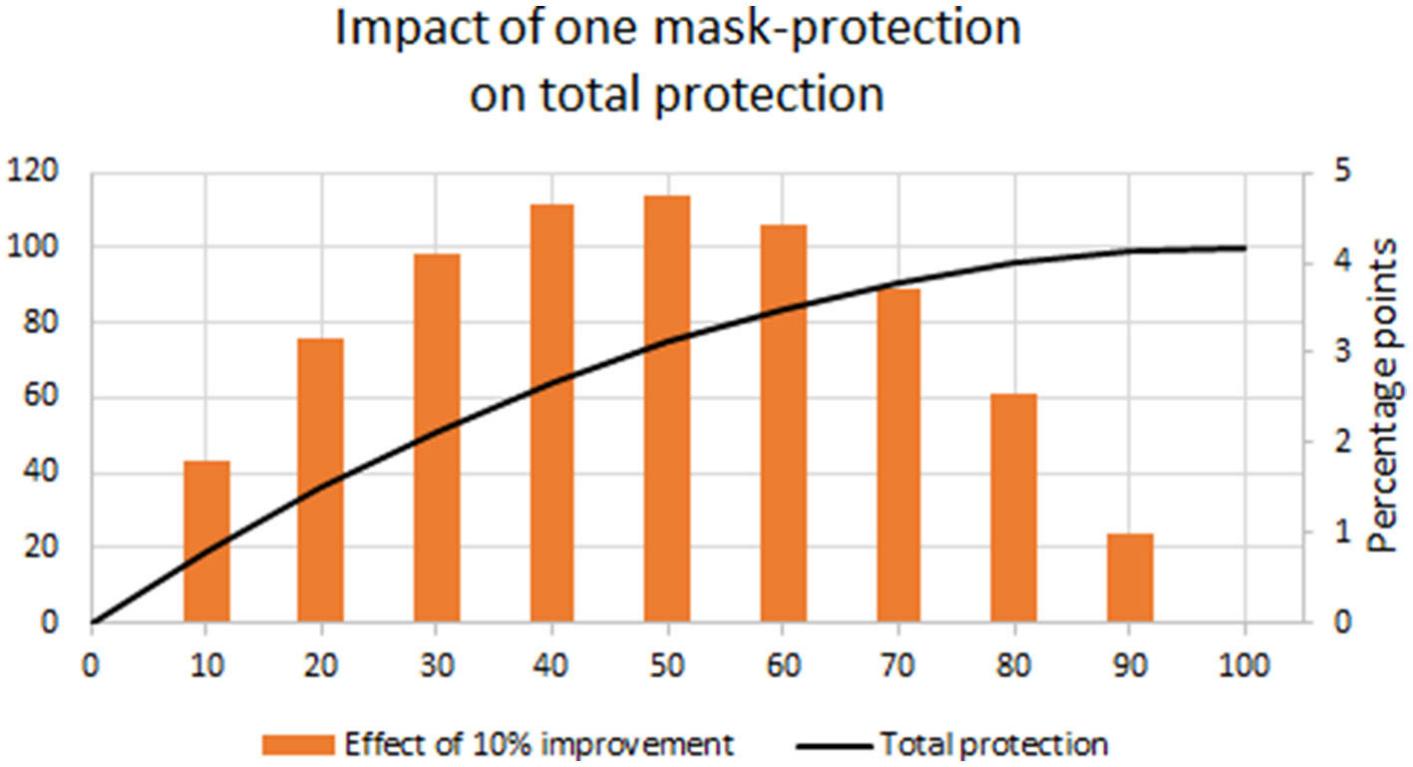
Total protection in % (by masking an infected and a healthy individual who are in contact) (orange line, scale to the left) and marginal utility of increased one mask-protection as a function of one mask-protection. The gray bars with the scale to the right show the effect on total protection (in percentage points) of a 10% increase in one mask-protection.

An important consideration that is often not mentioned when masks are discussed is the fact that they enable two barriers to be raised between an infected and an uninfected person. Masks worn simultaneously by infected and uninfected individuals would be expected to compound the reduction of transmission as follows: If the protection of one mask (“one mask-protection”) is x, and it is assumed that the size of the protective effect is the same for infected and uninfected persons, the total protection is (1-(1-x)^2^), illustrated in Figure 1 (solid orange line). This would amplify the protection afforded by the masks, and if an infected individual does not wear the mask properly, the masks of uninfected individuals nearby will offer some degree of protection, and vice versa. It should be noted that masks can be worn by infected individuals to protect other people or by uninfected individuals to avoid respiratory pathogens in their surroundings. Publications do not always make clear the distinction between output protection (from the former situation) and input protection (from the latter situation). Output and input protection may differ, and there is evidence that mask on source is often more effective than mask on receiver (15).

### Transmission of Respiratory Pathogens Through Masks. Different Ways to Measure Transmission and Protection

The protective ability of a mask can be expressed and measured in different ways, but is not always discussed in a comprehensive manner. The share of virions (the individual virus particles that can be visualized by, e.g., electron microscopy) or other particles that pass through a mask is often termed “penetration” and determined as the ratio between the concentrations inside and outside the mask. The “efficiency” of a mask is a measure of how much of the agent is turned away by the mask and how much has 100% penetration (16).

**Figure 2:**
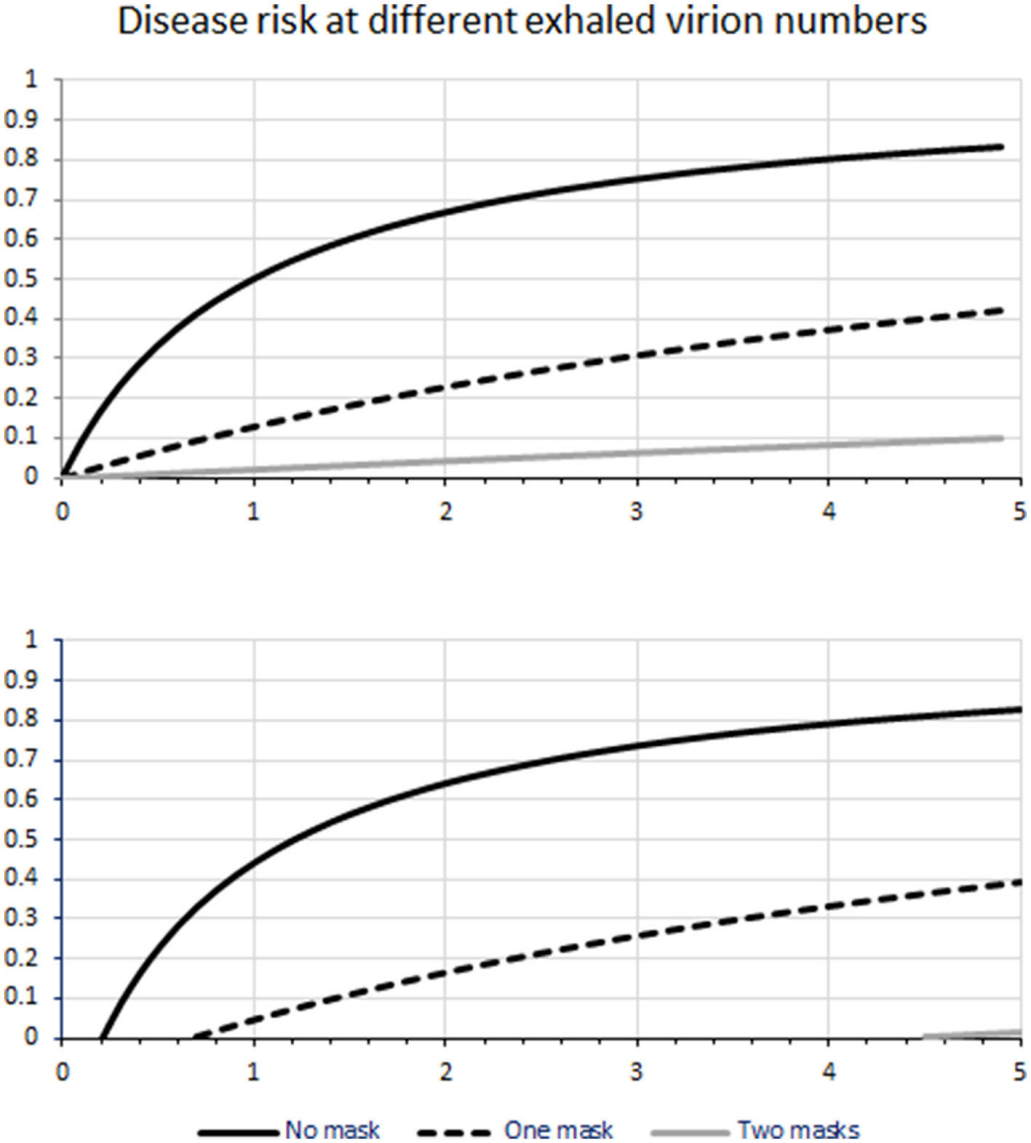
A sketch that attempts to illustrate the transmission of respiratory pathogens through masks worn by infected virus sources and uninfected recipients. The calculations assume a simple bimolecular interaction between the virus and its receptors. The units on the horizontal axis are arbitrary. The vertical axis shows the likelihood of being infected. Notice the short line in the lower right of **(B)**; this figure concerns two mask-protection in case there would be a minimally infectious dose well above 1. **(A)** shows the situation in the absence of a minimal infectious dose.

Notice that at very high concentrations of virions (to the right in Figure 2), there is so much excess of virions received that the number of virions received becomes less important and therefore the risk reduction by one or even two masks becomes small. On the other hand, there might be a minimal infectious dose of virions, below which infection does not occur. This could mean that a small reduction in virion count by mask use might completely abolish infection. However, the existence of a minimal infectious dose is under debate, it may be situation-dependent, and for some viruses, the minimal infectious dose may be equal to 1 (17). Of possible relevance here is the curious observation that a high percentage of morphologically identical viral particles in a sample, as determined by electron microscopy, will often be non-infectious. This observation may turn out to be of great importance, as it means that virus particles detected by some methods might be non-infectious. To the author, this suggests that there are inefficiencies in virus production and storage that can be exploited, see below. In line with this, infectious penetration through a barrier can be lower than the physical penetration of virus particles, see below. There is evidence for a minimum infectious dose of MERS that is well above 1 (18). If this is the case for SARS-CoV-2, it might work together with the synergistic effect of two masks in series to multiply the number of virions needed to cause disease (Figure 1). A dose-response (illness) curve has been published for SARS (19).

### Spread of Respiratory Infections: Droplets, Aerosols, and Other Routes

Several routes and modes exist for the transmission of respiratory infectious diseases; droplets may contribute to several of them. The modeling by Stilianakis and colleagues divided droplets into respirable droplets, with droplet diameters <10 μm, and inspirable droplets, with diameters in the range 10–100 μm. According to these authors, droplet dynamics is determined by their size, whereas population dynamics is determined by, i.a., pathogen infectivity and host contact rates. Robinson et al. of the team just mentioned (20) suggested that small droplets (~0.4 μm) have too small a viral load to be significantly infectious and that larger droplets (~4 μm) are the primary vehicle for infection. For SARS-CoV-2, a recent publication (21) argues from global trends in the number of infected individuals that airborne transmission is the dominant route, among several. Tellier et al. (22) has argued that SARS-CoV and MERS-CoV (viruses that cause SARS and MERS) may have to penetrate directly into the lower respiratory tract before causing disease; these authors also note that terminology in this area is not uniform. Whether an infectious agent is transferred by large droplets or airborne/aerosol may be important to the choice of protective equipment, as there is evidence that a conventional surgical mask is insufficient to protect against aerosol transmission, and that more elaborate masks may be more appropriate. It has been suggested that it can be transferred thorough the eyes [see (23)]. If this is a major route, the effect size of mask intervention would be smaller than filter penetration indicates, but eyewear would optimize protection.

### Estimates of the Protection Offered by Masks

A recent literature review found evidence that simple cloth masks reduce virus spread, despite their meshes being larger in size than the virus as well as aerosol droplets (24). It was found that under unfavorable conditions, more than 3% of MS2 virions penetrated through filters of N99 and N95 respirators (25). Wiwanitkit and collaborators (26) found that the size of the pores of the N95 mask is about 300–500 nm in diameter whereas the size of the avian flu virus is about 100 nm (SARS size may be similar), i.e., 3–5 times larger than the virus and there is evidence that SARS can pass through N95s (27). Simple fabrics were reported to have 40–90% instantaneous penetration levels of polydisperse NaCl aerosols, much worse than for N95 respirator filter media. In addition, N95 masks also have about a 10% leakage problem around the mask. The study by Rengasamy et al. (28) suggests that the upper level of efficiency for the common N95 mask may be 85%, i.e., 10% leakage on the side and 5% penetration through the filter. At the velocity of coughing, one team found an about 50% filtration efficiency of ultrafine (0.02–0.1 μm) particles by factory-made masks (29).

As most studies on masks have been carried out with other pathogens than SARS-CoV-2, a pertinent question is whether the size of the protection of masks is similar for different respiratory pathogens or not. This is addressed in the work of Zuo et al. (30) which indicates that the efficiencies of masks for excluding different pathogens may be similar. Eninger (31) concluded that studying different masks to measure the penetration of simple NaCl aerosols may generally be appropriate for modeling filter penetration by virions.

Balazy et al. found evidence that different models of the same type of mask can have very different protective properties (25). Zuo et al. (30) found that although physical penetration of adenovirus and influenza virus aerosols through respirators can be substantial, 2–5%, infectivity penetration of adenovirus was much lower. The meta-analysis of Offeddu et al. (32) quantified the protective effect of face masks and respirators against clinical respiratory illness (risk ratio [RR] = 0.59) and influenza-like illness (RR = 0.34). Meta-analysis of observational studies provided evidence of a protective effect of masks (odds ratio OR = 0.13) and respirators (OR = 0.12) against severe acute respiratory syndrome (SARS).

Dbouk and Drikakis (33) presented a fluid dynamics study of the transmission of respiratory droplets through and around a face mask filter during coughing. These authors showed output (mask on infected individual) as well as input (mask on healthy individual) protection but the protection was limited to perhaps 80–90%. The systematic review by Jefferson et al. did not show a clear reduction in respiratory viral infection with the use of medical/surgical masks during seasonal influenza; the selected papers did not allow definite conclusions (34). However, Taiwan reportedly quickly eliminated their COVID-19 outbreak using a combination of interventions that included masking (35). The recent meta-review by Violante and Violante (36) suggests that surgical masks and N95/FFP2 respirators protect equally well-against airborne viral infections. The review just mentioned is also useful in that it summarizes the requirements for different classes of masks including differences between European and US standards, and that it lists some references in the field that are not cited directly in this paper. A German review found only weak evidence for masking being efficient as a hygienic tool to prevent virus spread, but better evidence for close contact scenarios. This review emphasizes potential risks (37). A number of different routes exist for SARS-CoV-2 transmission, some of them involving the eye (23). In the calculations of Sewell et al. (38), the estimated effect of the face mask *mandate* was a reduction in transmissibility (pt) of 23%. The effect of *masking* was presumed to be larger than this, because some individuals wore masks in the absence of a mandate. Cases selected here for closer study are shown in Table 1.

**Table 1:** Four cases representing four different levels of protection by mask that have been considered in this study and incorporated into the simulations that use COVID-19 Scenarios.

**Case no**.	**Pathogen removal/risk reduction, one mask**	**Removal/reduction, two masks**	**References**
1	99.7%	99.9991%	Ref. (14). An advanced mask.
2	85%	97.75%	N95 mask. This is estimated from data in (15), assuming 5% filter penetrance and 10% leakage on the sides.
3	22%	39.16%	Average of input and output protection by a simple home-made mask; based on measurements in (39).
4	5.7%	11%	Based on the 0.89 relative risk reported (40) in a meta-analysis of Hajj pilgrims. Notice that in some segments of the population studied, actual mask use was <50%.The question to be addressed here is whether masks can influence the epidemic even if many do not use their masks properly.

*Notice that although most of the numbers in this Table are taken from published papers, they may not be representative for all pathogens or varieties of masks. None of the numbers in the table are derived from a study on COVID-19*.

### Calculations and Simulations to Address Early and Late Interventions

This study attempts to estimate the effect of masking the general population in a COVID-19 outbreak, first using simple considerations about the basic reproduction number of the epidemic and the level of protection from different types of masks. The basic reproduction number (R_0_), is an index of the contagiousness of an infection and depends on both the infectious agent and other factors. As R_0_ is the expected number of secondary infections produced by an index case in a completely susceptible population, it is often used to predict if an outbreak is expected to continue, as R_0_ >1 indicates that it will and R_0_ <1 indicates that it will not. However, this is a simplification and the calculations surrounding R_0_ can be complex (41, 42). After gathering some published numbers regarding the R_0_ of the COVID-19 epidemic and the protection afforded by masks, this study then moves on to simulations of the effect of masking with three separate types of software, one of them a specialized COVID-19 program and another one a simpler program intended for educational purposes.

Then the effect is addressed using computer simulations. Claims are sometimes made that disease-preventive measures must be enacted early in order to be effective. Early intervention has been claimed to be important also regarding COVID-19 [for example (43, 44)]. Both early and late intervention was simulated using the computer software.

### Non-linear Systems Often Show Behavior That Is Not a Simple Function of the Size of a Disturbance or an Intervention

Since the initial results of this study were presented in a seminar and preprint form in the spring and summer of 2020, the finding that masks might have a dramatic effect on COVID-19 has sometimes been met with skepticism. A question asked repeatedly by colleagues is “How can the effect of an intervention be larger than the size of the intervention even to the point of eliminating an outbreak?” A related question sometimes asked is “How big an effect of an intervention such as masks is enough?” Spatial aspects have been brought up by suggestions that masking may be unnecessary in less populated areas [e.g., by Noren in a comment to (45)]. The questions just mentioned are now addressed here by considering a system of interconnected individuals. Highly interconnected systems can result in non-linear effects, e.g., in infection spread, as shown by Heesterbeek et al. (46). In the face of such complexity, mathematical models might aid the understanding of patterns of spread. The paper just cited also points to the existence of paradoxical effects, i.e., an intervention can sometimes have opposite effects depending on the state of the system.

In nervous system functioning as well as infection spread, units (neurons or persons) receive inputs (virus loads or synaptic activity) from many other units; when a threshold is attained (e.g., becoming infected or reaching firing threshold), the nodes distribute their activity back to the network of units (neurons/individuals) by releasing virions or neurotransmitters. Thus, a wave or cascade may move through the system, sometimes described mathematically as an avalanche. Systems prone to avalanches are often said to be in a critical state (47), i.e., within a narrow margin between avalanche propagation and extinction (48). A related but distinct concept is bifurcations, which are more applicable than critical points for certain systems but also indicate states at which a small change in one parameter can dramatically change the functioning of the system. Excitation/depression waves in interconnected networks can be very sensitive to parameters and be controlled by weak external forces (49). Varying a parameter such as synaptic strength (corresponding to transmissibility in infectious epidemiology) can at bifurcations select between dramatically different states of the system (50).

Despite many similarities, a difference between networks of neurons vs. networks of disease-prone humans is that neurons can normally fire nerve impulses many times, whereas a person will often have the same infection only once. This difference between neuronal networks and infectious network is not all-or-none, since neurons can have refractory periods or fatigue during which it is impossible or difficult to elicit a neuronal impulse. On the other hand, it is known that coronaviruses including SARS-CoV-2 can cause re-infection (51).

The programs COVID-19 Scenarios and Epidemix (detalied below in Materials and Methods) do not explicitly model the spatial aspects of infection spread and have turned out to be difficult to understand by some. A third software is therefore introduced in this paper as an attempt at simulate and visualize effects of imperfect interventions such as masks on infection spread in space using network simulation. Unfortunately, computer programs for network simulation that are easy to use are often very limited in scope and those that are flexible may require experience with computer programming and with a particular piece of software. Simbrain 3.0 (52) was chosen for this paper and is a program for the computer simulation of brain circuitry whose graphical user interface speeds the creation of networks. It also allows rather large networks consisting of thousands of components to be built by the writing of scripts. Simbrain can especially visualize the internal states of a network.

Despite seemingly thousands of publications about masks in COVID-19, only a few of them are articles that make use of simulation. Aspects in this paper that are little covered or not at all in previous publications are how mask protection of infected and uninfected wearers and a hypothetical minimal infectious dose combine quantitatively to limit transmission, time effects on effect size, fundamental limits on the impact of masking, and possible routes to improved masks. Of importance in the design of the simulations of this paper is that two existing software programs documented in publications were used with a minimum of modifications to parameters in order to reduce bias.

## Materials and Methods

Since a commonly expressed opinion in the author's country was that masks in the general population were of little or no value, the author decided early on to use at least two separately developed and widely used pieces of software to arrive at a conclusive result regarding the effect, or lack thereof, of masking. At the time of inception of this study (early 2020), there were (at least) two major software programs for modeling the specific spread of COVID-19, COVASIM (53) and COVID-19 Scenarios (54). Since the web version of COVASIM (when tried by the author) offered less flexibility to input multiple parallel interventions, COVID-19 Scenarios was chosen. Epidemix 2 was used because of its simplicity, use in education in epidemiology, and because it is well-documented in publications. Simbrain 3.0 was used to begin addressing spatial aspects of COVID-19 spread, as there were large regional differences in COVID-19 cases and because it was proposed that masking could break chains of COVID-19 transmission. To avoid bias, software developed by others for computer simulations of a standard population level epidemiological models (such as COVID-19 Scenarios and Epidemix 2) were preferred, and default parameters of the software was used as much as possible.

As described in detail (54), COVID-19 Scenarios simulates a COVID-19 outbreak with a generalized SEIR model with the total population divided into age-strata (because of known age dependence of COVID-19 outcome) compartments of: susceptible (S), exposed (E), infected (I), hospitalized (H), critical (C), ICU overflow (O), dead (D), and recovered (R) individuals. People transition among the different compartments. The model allows researchers to specify individual interventions with start and end dates to model the existing (social distancing, case isolation, and quarantine) as well as additional interventions. COVID-19 Scenarios provides default parameters estimated from real-life statistics, although the authors emphasize the uncertainty behind these estimates. We ran the simulation for the United States. The model had been calibrated by its authors to match its age structure and the observed epidemiological statistics. The simulations did not consider saturation phenomena that might occur at very high virus counts (Figure 2), neither a possible minimal infectious dose of virions (Figure 2). It was assumed that transmission occurred only through routes that can be blocked by mask use.

Notice that although most of the numbers in Table 1 are taken from published papers, they need not be representative for all pathogens or varieties of masks. None of the numbers in the table are derived from a study with COVID-19.

The second software used in this paper is Epidemix 2 (55), which is a simplified software for teaching and demonstration purposes. It uses a visual interface to access eight models of epidemics without dealing with the details of mathematical equations and program code. The underlying calculations are done by a set of software packages in the programming language R. All models simulate disease spread through a population, allowing the user to select the model and characteristics of the population, interventions, etc.

Curve-fitting to estimate the slope of the curve of scenarios with different late mask interventions was done using the diagram function in Excel for Microsoft 365.

**Figure 3:**
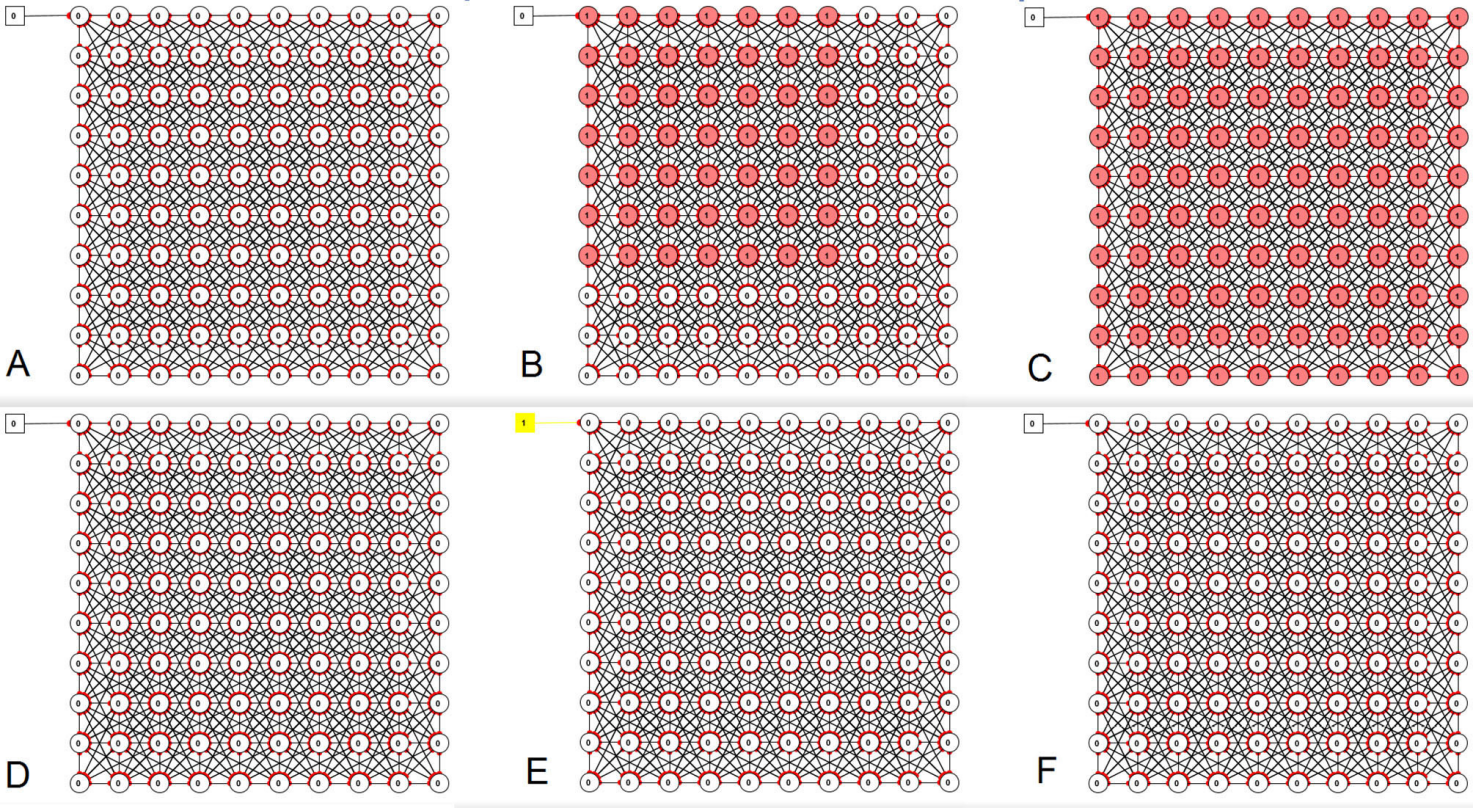
Use of Simbrain 3.0 to simulate infection spread among interconnected individuals. Notice that the current parameters have not been carefully calibrated against any particular real-life situation and should be considered rough first estimates used for illustration purposes. It can be seen that models can easily be found in which a relatively minor change in the transmissibility can have dramatic effects on the system. It can also be demonstrated that for many systems, which could be achieved with masking combined with a set of other interventions, it is impossible or almost so to achieve a wave of infection transmission in the system. Red color shows those nodes that are active (i.e., exhibiting neuronal activity of being infected). Upper row **(A–C)**: Coupling between neurons 10% increased to enable a wave of infections to move through the system. Lower row **(D–F)**: With 10% reduced coupling between individuals, it is very difficult to elicit a wave of infections in the same system, all other parameters are equal. **(A/D)**, **(B/E)**, and **(C/F)** show three sequential time points.

With the third piece of software, Simbrain 3.0 (52), a model was built of 100 nodes (representing persons or groups of persons) that can infect their nearest neighbors (shown by the oblique lines in Figure 3), but not beyond that. There are only excitatory connections. An impulse generator was added that enters activity (corresponding to virus loads) into the network through one of its nodes (in the upper left of the network) at random intervals. During simulations with this model, the effect of an increase in connection strength (i.e., transmissibility) of 10% as well as a 10% decrease in transmissibility is tested. Related work regarding the spread of HIV among interconnected individuals' social networks is described by Delva et al. (56).

## Results

### A Simple Estimation of the Degree of Protection Afforded by Masks and the Protection Needed to Influence the Epidemic

The transmissibility of a virus is measured by the basic reproduction number (R_0_), which measures the average number of new cases generated per typical infectious case. As described by Rahman et al. (57) and references therein, an R_0_ of 1.0 is an important threshold value. If the R_0_ is equal to 1 or less, this indicates that the number of secondary cases will decrease over time and, eventually, the outbreak will peter out. One review evaluated the mean and median of the R_0_ estimated by the 12 articles and they calculated a final mean and median value of the R_0_ for COVID-19 of 3.28 and 2.79 (58) in line with a recent review (59). This seemingly provides a rough indication of how much the transmission must be reduced to reverse the epidemic. It appears that a reduction of transmission of at least two thirds is necessary. This is within the range of protection of some but not all masks (some 67% efficiency). However, as there would be two barriers between infected and non-infected individuals, the numbers for two serially connected masks should presumably be calculated, as the mask of the uninfected individual will add to the protection from the mask of the infected individual, indicating that many of the available masks might be adequate.

### Simulations Using COVID-19 Scenarios Modeling Masks During the Start of an Outbreak

**Figure 4:**
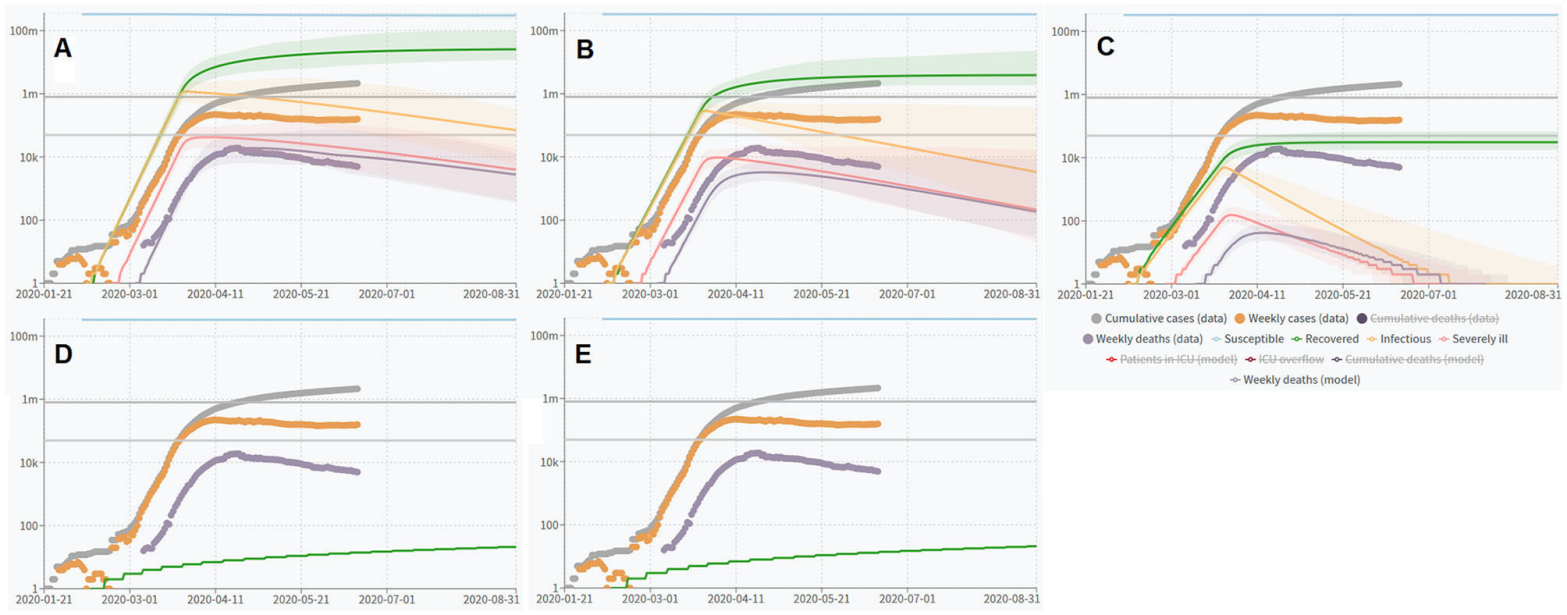
The effect of masking the general public on the COVID-19 epidemic, as estimated using a simulation by COVID-19 Scenarios. **(A)** No mask, **(B)** 11% protection; **(C)** 39% protection, **(D)** 97.75% protection, and **(E)** 99.9991% protection.

Figure 4 shows the simulated number of confirmed COVID-19 cases in the U.S. vs. date. The vertical axis is a logarithmic scale. The linearity of this graph shows the exponential growth expected early in an epidemic. We ran the simulation for the period ending with August 31, 2020. This was long enough to see the effects of masks on the time course of the epidemic. The default parameter values of COVID-19 Scenarios, used as input to this program, with mask interventions added as indicated in the text, are shown in Table 2. We wanted to observe the sensitivity of the outcomes to the time when mask intervention was started, rather than the effects of different mitigations implemented at the same time.

Table 2Parameters used for COVID-19 Scenarios.

**Table:** 

**Scenario: United States of America (edited)**
**Parameters**
Population
Parameter	**Value (summer 2020)**	**Value (December 2020)**
Age Distribution Name	United States of America	United States of America
Case Counts Name	United States of America	United States of America
Number of hospital beds	798288	798288
icu Beds	49499	49499
Cases imported into community per day	0,1	0,1
Number of cases at the start of the simulation	1	1723495
Population size	327167434	327167434
Seroprevalence		14,95
Epidemiology
**Parameter**	**Value**	**Value**
Hospital Stay Days	3	7
icu Stay Days	14	14
Infectious period Days	3	3
Latency Days	3	3
Increase in death rate when ICUs are overcrowded	2	2
Seasonal peak in transmissibility	January	January
RO at the beginning of the outbreak	4.1–5	4.1–5
Seasonal variation in transmissibility	0	0

**Table:** 

Mitigation [added to the default values]:	Intervention 1									
**Reduction of transmission (mitigation included in default parameters of COVID-19 Scenarios summer 2020)**
Mar 24 2020–Sep 01 2020	73.8–84.2%	
**Reduction of transmission (mitigation included in default parameters of COVID-19 Scenarios December 2020)**
Jan 07 2020–Feb 12 2020	78.3–87.7%	
Feb 12 2020–Mar 04 2020	19.6–22.4%	
Mar 04 2020–Mar 26 2020	23.1–26.9%	
Mar 26 2020–Apr 23 2020	67.2–78.8%	
Apr 23 2020–May 14 2020	70.5–81.5%	
May 14 2020–Jun 08 2020	68.3–79.7%	
Jun 08 2020–Jul 12 2020	63.1–74.9%	
Jul 12 2020–Aug 08 2020	69.4–80.6%	
Aug 08 2020–Sep 05 2020	70.5–81.5%	
Sep 05 2020–Sep 29 2020	67.2–78.8%	
Sep 29 2020–Nov 09 2020	64.1–75.9%	
Nov 09 2020–Jan 11 2021	66.2–77.8%

**Table:** 

Mitigation [added to the default values]:	Intervention 1									
**Reduction of transmission**		
Mar 24 2020–Sep 01 2020	73.8–84.2%	

*These were the default parameters provided by the software when the simulations were done. Please notice that the defaults in the summer of 2020 included a 73.8–84.2% intervention introduced on March 24, that is included with the software. When mask interventions were included in the simulation, they were introduced on January 1 (i.e., for the whole period of the simulation), July 1, or (for simulations run in December, 2020) on December 1*.

The results from the first runs with COVID-19 Scenarios show that mask use appears to be effective even at low one mask-protection or limited compliance. Even the lowest efficiency scenario reduced the simulated epidemic if applied from its beginning. The two highest protective abilities seem to practically completely abolish the epidemic. There was no indication of any difference between the two masks with the highest protection modeled (scenarios 1 and 2). It appears that even simple masks (e.g., 21% protection, scenario 2) or low-compliance mask-wearing (11% protection, scenario 1) reduces the number of COVID-19 cases within weeks.

### Simulations Using a Simplified Model Using Epidemix Version 2

**Figure 5:**
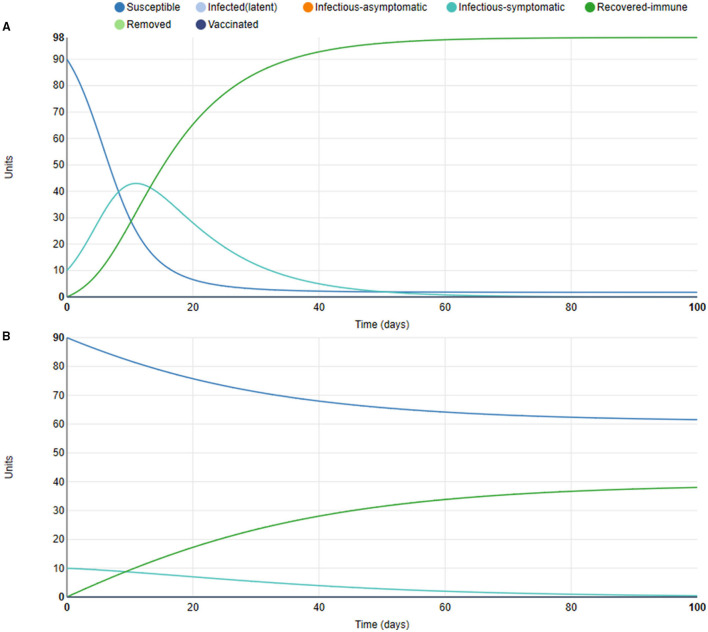
Simulations using Epidemix version 2. **(A)** 10% infected initially, otherwise full defaults of the Epidemix program. **(B)** 10% infected initially, 75% two-mask protection, otherwise full defaults of the Epidemix software.

These simulations used the full defaults of Epidemix version 2, i.e., the parameters were not specifically calibrated for SARS-CoV-2 (deterministic homogenous model, infection states S, Is, R, population size: 100, daily number of effective contacts per unit: 0.4, length of infectious period: 10 days; Figure 5A). An added intervention with 75% two-mask protection, corresponding to a simple mask, was tested by reducing transmission by 75% (Figure 5B).

As shown in Figure 5, the cumulative number of infected individuals during the whole simulation was only half in the mask use scenario as compared to the default scenario, with the effect of masking present throughout the period. The result seemed to corroborate the result from the COVID-19 Scenarios model that masks reduce COVID-19 cases.

### Do Interventions Have to Be Applied Early?

**Figure 6:**
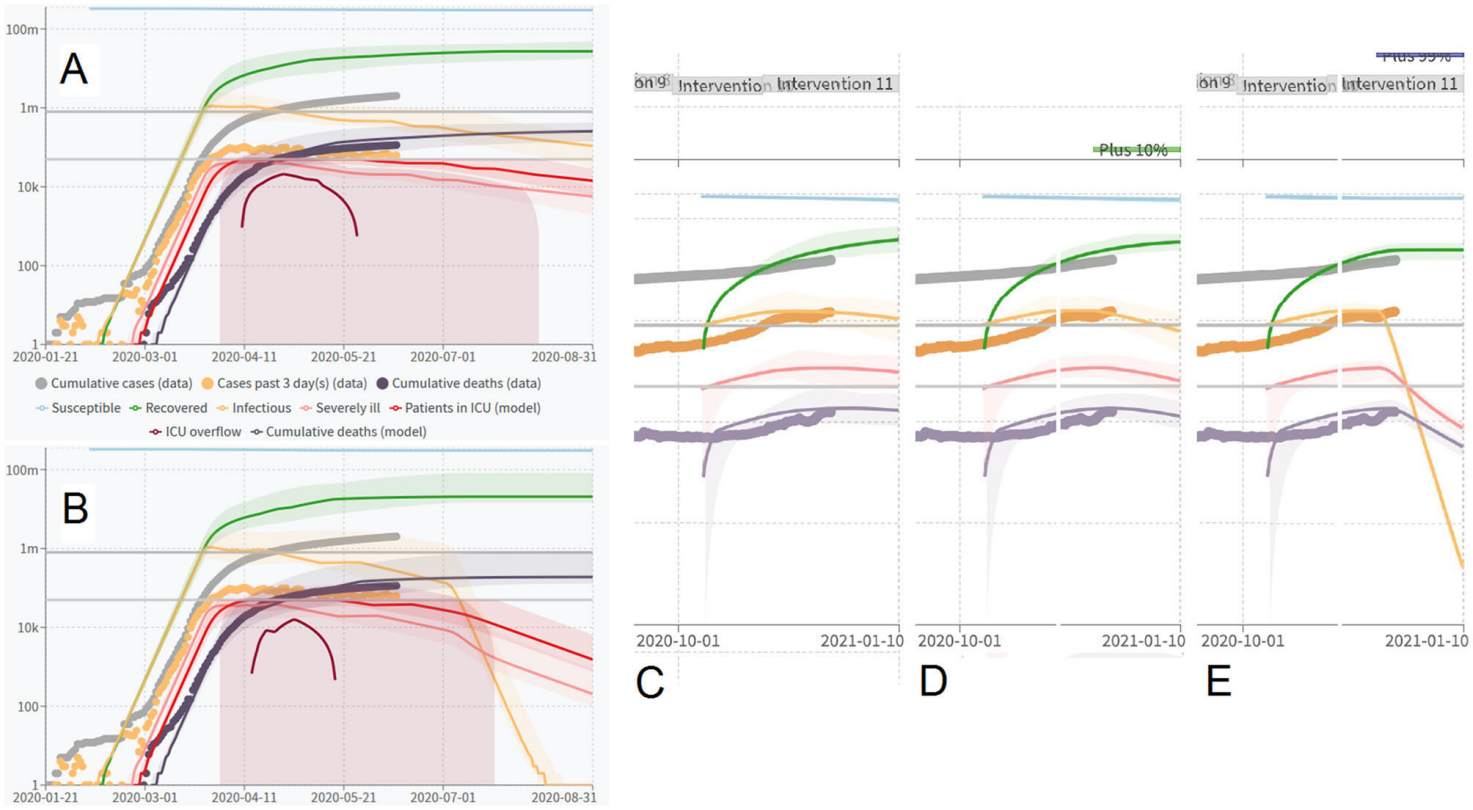
Effects of late mask interventions on the number of active COVID-19 cases. **(A)** Full defaults according to COVID-19 Scenarios. **(B)** Full defaults with late mask intervention using advanced mask (99.9991% two-mask protection). **(C–E)** show the effects of interventions beginning on December 1, 2020: **(C)**, full defaults; **(D)**, the effect of an added 10% protection, and **(E)**, the effect of an added 99% protection intended to approximate the effect of population-wide wearing of SCBA devices.

**Figure 7:**
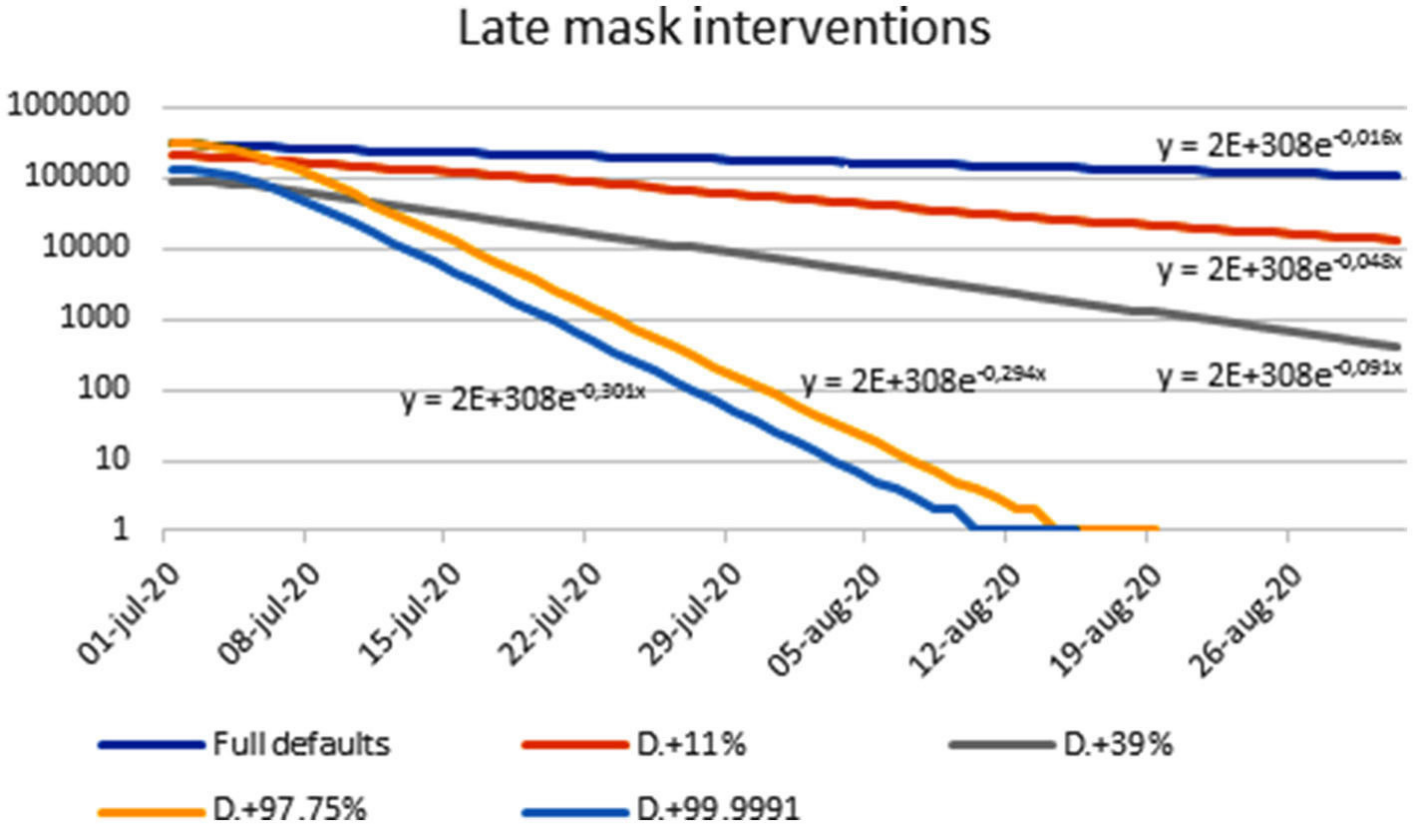
Estimating half-life and weekly reduction from the late use of four different masks (cases 1–4); vertical axis is “infectious” individuals from COVID-19 Scenarios.

From the results in Figures 6, 7 that shows curve-fitting of the data in Figures 6A,B for July and August 2020 only, it seems that they do not. When they are applied late, the total number of cases is influenced less than active cases, since a mask will not help individuals who have already been infected. However, the number of active cases is reduced in all mask interventions modeled, with some interventions resulting in a dramatic reduction. With case 4, in 11% two-mask protection, the reduction in new cases was about 11% per week. With case 1, reduction was quite dramatic at approximately 88% per week.

An attempt was made to estimate the effect of population-wide masking in a situation when mask use was already high at the end of 2020 (Figures 6C,D) by adding an extra intervention of 10% to the defaults using the latest version of C19S. The effect of a virtually ideal intervention such as SCBA was also estimated (Figure 6E).

### An Attempt at Simulating and Visualizing Effects of Imperfect Interventions Such as Masks on Infection Spread Using Simbrain Version 3.0

Using Simbrain, a network of 100 interconnected nodes was set up, feeding stimulating input (representing virions) to each other (Figure 3), originating from an impulse generator (upper left). During simulations with this model, the effect of a small increase in connection strength (i.e., transmissibility) of 10% as well as a 10% decrease in transmissibility was found to produce dramatic differences in the behavior of the interconnected system under some conditions. This shows that an imperfect intervention of about the magnitude of a simple mask, can at times have a dramatic effect on an interconnected system. Videos exemplifying the output of Simbrain are included in the Supplementary Materials.

## Discussion

The results seem to indicate that it is possible to identify timepoints and situations at which an intervention of limited size, e.g., masking, will have a major effect on a COVID-19 epidemic and situations at which the wearing of masks is of little help, e.g., where there is a big excess of virions. Given the well-known propensity for “bugs” in large computer programs (presumably including those in epidemiology), this author thinks that publishing simulations with multiple software programs and by multiple, independent research teams is necessary. While it may be unrealistic to equip the whole world with SCBA equipment, widespread use of such equipment seems (even in locations with excess virions) likely to clear a circumscribed area of SARS-CoV-2 contagion. Modeling also seems to be of value for education on the spread of infection and the effects of masking. The effect size of masking may vary even more than from the different performance of different masks modeled above, due to differences in compliance and fitting of the mask, other routes of infection, pre-existing immunity but also because a system of interconnected, disease-prone individuals may have non-linear properties. As even the smallest mask intervention had a long-term effect in the simulations, the simplest mask could result in a large reduction in deaths if intensive care units are working close to capacity. That the effect size was found to compound over time is reminiscent of interest-on-interest in a bank account, when the interest becomes sizable after being applied repeatedly. When mask protection >50% was simulated, the effect on the size of the epidemic was dramatic, from about 1 million to zero fatalities (Table 3).

**Table 3:** Data from COVID-19 Scenarios regarding the number of individuals in the different compartments of the model (simulation performed summer 2020).

**Time 2020–08-31**	**Cumulative recovered (total) median**	**Cumulative recovered (total) lower bound**	**Cumulative recovered (total) upper bound**	**Cumulative hospitalized (total) median**	**Cumulative hospitalized (total) lower bound**	**Cumulative hospitalized (total) upper bound**	**Cumulative ICU (total) median**	**Cumulative ICU (total) lower bound**	**Cumulative ICU (total) upper bound**	**Cumulative fatality (total) median**	**Cumulative fatality (total) lower bound**	**Cumulative fatality (total) upper bound**
Default	25435572	11699016	100463893	892376	410277	3528347	468735	218699	1754633	224006	97251	996242
Default + mask 11% (scenario 4)	3879172	1071446	10906385	136042	37572	382708	73074	20270	200586	33934	9498	89024
Default + mask 39% (scenario 3)	30719	17446	67805	1078	612	2378	581	330	1282	273	155	600
Default + mask 97.5% (scenario 2)	21	21	21	1	1	1	1	1	1	0	0	0
Default + mask 99.9991% (scenario 1)	21	21	21	1	1	1	1	1	1	0	0	0

As the calculations illustrated in Figure 6 and Table 4 suggest that complete elimination of COVID-19 can be achieved in a closed community with <2 months of intervention with highly protective masks, it would be of interest to identify a community suitable for a clinical trial with such masks. Any such study should be carefully carried out taking local conditions (incl. legislation) into account and use continuous evaluation of infection parameters as well as any side effects of the masks. It should be said that the results above seem to be robust, as the principal results do not seem to depend on the precise model used or its input parameters, as slight variations of some parameters that have been tested have not altered the fundamental results.

**Table 4:** Estimates of T_1/2_ of “infectious” individuals with different late mask interventions.

**Intervention**	**Multiplier in exponent estimated from graphs**	**Half-life**	**Weekly reduction in active COVID-19 cases (%)**	**Data in figure**
Default values only	−0.016	43.3	11	6A, 6B, 7
Case 4	−0.048	14.4	29	6A, 6B, 7
Case 3	−0.091	7.6	47	6A, 6B, 7
Case 2	−0.294	2.36	87	6A, 6B, 7
Case 1	−0.301	2.30	88	6A, 6B, 7
Default values only (includes some masking)	−0.008	86.6	5	6C-E
10% extra reduction in transmission presumed to result from everyone wearing masks	−0.024	28.9	15	6C-E
99% extra reduction presumed to result from everyone wearing SCBA apparatus	−0.31	2.24	89	6C-E

### Side Effects and Risks of Masks

There may be few comprehensive studies of side effects of masks. A recent systematic review found that side effects of masks were rarely measured in studies of masks against respiratory disease (34). From everyday experience, side effects and risks of mask use are usually limited to minor discomfort. *Skin problems* are among the most common side effects of masks. Pain and pressure from masks have been described during the COVID-19 pandemic, and remedies have been suggested (60). Gefen et al. studied device-related pressure ulcers in the context of COVID-19 (61). Ju et al. reported contact vitiligo from rubber ear loops from a mask (62). Contact dermatitis due to masks and solutions are described by Altobrando (63). Chaiabutre found skin reactions in persons wearing masks during the COVID-19 pandemic (64). Protruding ears in children has been mentioned as a possible side effect of masking (65). However, serious *accidents* from some masks may be possible: One of

the more serious side effects of mask-wearing seems to be that some masks may cause increased condensation on eyeglasses when worn concurrently (66), potentially obstructing the view of the wearer; however, counteracting devices are known. Heavy respiratory protective equipment used by firefighters can affect the balance of the user (67), potentially increasing the risk of fall accidents. *Effects on breathing and blood gases* have been described: Santos-Silva et al. (68) point to increasing resistance to air intake, lowering airflow into lungs, and causing temporary reduction in breathing rhythm. There may be symptoms related to hypercapnia (69)–breathing and gas exchange (70). A German review found evidence for significant respiratory compromise in patients with severe obstructive pulmonary disease, secondary to development of hypercapnia and possibly lung infections which emphasizes their potential risks (37). Kyung and coworkers (71) found that subjects with COPD found increased dyspnea, breathing frequency, and blood oxygen saturation after N95 use. It was suggested that N95 masks should be used with care in patients with more severe COPD. It was pointed out that lint and fibers from textiles are known to contribute to lung problems when inhaled in large quantities (29); it seems unclear how much this applies to masking during an epidemic. *Psychological effects* have been described: The review by Perna suggests that persons prone to panic attacks might experience discomfort due to increased respiratory physiological burden in RPD wearers' increased breathing resistance, CO_2_ rebreathing due to CO_2_ accumulation in the RPD cavity, and decreased inhaled O_2_ concentration (72). King has suggested that mandatory mask-wearing may give rise to difficulties in emotional communication due to impaired communication of facial expressions (73). Masking has been found to impair identification of faces both by human observers and face recognition computers [(74) and references therein]. One study found that mask reuse and use were associated with depression (75). *Survival and growth of microorganisms and spread of infection* have been reported: Bacteria can survive on surfaces of masks for several days (27). Personal protective equipment was reported to be a source of airborne infections (66). Bamber and Christmas (76) and others have pointed out that discarded masks may be a biohazard and must be discarded of properly. *Reduced physical activity* has been reported during mask-wearing (77). This section does not attempt to be a complete list of possible side effects of masks.

### Possible Avenues for Improved Masks and Masking

There is scope for improvement of masks. It has been shown that it is possible to improve the protection against a certain class of infectious agents (e.g., chemical treatments) or generally (for example, improving the fitting of the masks against the face of the wearer). 3D printing is beginning to be used to compensate for the shortage of personal protective equipment including masks (78), and could be used with existing knowledge about mask fitting [e.g., (79, 80)] to fit masks to individual anatomy. Furthermore, in situations when there is solid evidence for the value of masking, educational activities could educate the public about this value and help with the selection, purchasing, fitting, use, and disposal of masks.

Viruses have unique biophysical properties including elasticity/deformability, brittleness/hardness, material fatigue, and resistance to osmotic stress (81) that might be targets for antiviral interventions, perhaps also to produce better masks. An *antimicrobial surface* contains an antimicrobial agent that inhibits the ability of microorganisms to grow on the surface of a material. For example, it was shown that surfaces that are simultaneously hydrophobic and oleophilic have quicker deactivation of enveloped viruses (82) (which include SARS-CoV-2, although influenza A was studied). Another example is that copper and its alloys destroy a wide range of microorganisms. Technology can permanently introduce copper oxide into polymeric materials that are biocidal. Masks filtered above 99.85% of aerosolized influenza and H9N2 virus, and infectious influenza virus could be recovered from the copper-modified masks (83). Quan and colleagues (84) tested functionalization of a surgical mask with a sodium chloride salt coating that dissolved on exposure to virus aerosol and recrystallized during drying; such filters showed better filtration than conventional masks and all mice survived influenza virus penetration through the rough salt-coated filters. It has been proposed that purposefully engineered materials with nanostructured surfaces can eliminate enveloped viruses such as SARS-CoV-2 i.a., on masks and be self-disinfectant (85). Other ways in which *materials technology* can improve masks is exemplified by a mask under development that lets some SARS-CoV-2 penetrate the mask, but heat-inactivates it (86). Guha et al. suggest that electret materials (i.e., those with a permanent electric charge) may be advantageous for masks (87). Relatively few studies with aerosols for specific illnesses seem to exist. It should be valuable to conduct such studies before an airborne epidemic hits next time.

### Questions for Additional Research

In several studies, leakage on the sides of the mask has been greater than filter penetration of an agent. Therefore, efforts to improve fit or educate the public about proper mask-wearing are likely to be effective. The conclusion is that masks should be evaluated as an important addition to other ways of protection. They may have protective effects on the same order of magnitude as vaccines, but with the added advantages of being effective against a wide range of respiratory pathogens and can be prepared in advance and stored.

*Questions that can be addressed in future research on masks* include first and foremost a systematic study of side effects and risks associated with masks. Other research on masks would be a cost-benefit analysis to decide what level of protection is best when costs and side effects are considered. The gray bars in Figure 1 show the marginal improvement in two-mask protection from a 10% improvement in one mask-protection and indicates that the marginal benefit may be biggest from raising protection above 50%, and less benefit may be received from improvements in one mask-protection beyond 90%. It may be valuable to study masks with aerosols for specific illnesses before an airborne epidemic hits next time. Other research on masks would be to evaluate the shelf-life of different materials used in masks to select materials that allow for long-term storage preparation for future epidemics. Procedures for industrial and home manufacturing could be optimized. How to best educate individuals about the value of masks, how to properly wear a mask and perhaps how to make their own mask, and how to increase adherence to mask use are important questions to ask.

*Questions that can be addressed regarding interactions with other interventions* include do masking and social distancing work synergistically or do they work best on their own? In the simulations above, it implied that masks and other interventions depress SARS-CoV-2 transmission independently, but this is not necessarily the case in real life. Does the introduction of masks reduce people's compliance with other preventive measures, or do masks serve as a reminder of the epidemic, improving compliance with other measures? Can groups with low vaccine response [e.g., possibly those in (88)] be identified and should they wear a mask instead?

*Questions that can be addressed in future research on SARS-CoV-2* (and other pathogens) would be to confirm and quantify different ways of transmission. Such information would inform decisions about whether a mask is of any value and in which situation it may be of value.

The results from these simulations are encouraging, but the only way to be sure about the effects of masks is to conduct prospective, controlled studies; perhaps along the lines of Lin et al. (89). Also, masks and related equipment are associated with significant side effects and risks that should be carefully monitored during any implementation. The numbers used here are consistent with the literature but do not represent the whole literature and many numbers are derived from studies of other agents than SARS-CoV-2. The work described here may still be relevant for SARS-CoV-2 as using slightly different input parameters or slightly different models typically did not change the outcome of a simulation much. During the past several months, many publications have come out in favor of mask use by the public [e.g., (21, 90–93)]. Stutt et al. (94) using somewhat different modeling from ours found parameter ranges in which mask compliance and effectiveness could reduce the R0 enough to slow or stop COVID-19 spread.

The use of simulation may help decide whether a small reduction of transmission with a simple mask is sufficient. It may also help identify situations when an advanced protective device is needed such as an advanced mask or even an SCBA device. A major general question is to identify all pathways for COVID-19 transmission and their relative importance. Alternative pathways will reduce the effect size of mask interventions but are likely amenable to interventions parallel to masking. If an alternative pathway is major, its obliteration may be necessary to achieve the “corona washout” suggested above. From the findings of this study, to optimize a mask intervention, it seems necessary for a government to recruit suitable competence in technology, mathematics, human behavior, and risk management, and to consult with community and religious leaders. For life-changing events such as funerals, trials with masking (or perhaps even with advanced equipment such as SCBA) and contact-tracing can be considered, to arrive at a protective equipment that avoids most infections while retaining the aspects of the ceremony as much as possible. Simulation can give a glimpse of what protection can be achieved and help in the design of such trials. If mask measures, as suggested here, are put into action, it will be important to generate hypotheses that can be tested to see if the actions really work and are cost-effective. Here various modeling strategies may be very helpful. For example, the concentration of virus could be measured in public places with and without the use of masks.

## Data Availability Statement

The raw data supporting the conclusions of this article will be made available by the authors, without undue reservation.

## Author Contributions

The author confirms being the sole contributor of this work and has approved it for publication.

## Funding

This work was funded in part by internal funds of Stockholm County Council and Karolinska Institutet [Agreement concerning research and education of doctors (ALF) and the Research Funds of Karolinska Institutet].

## Conflict of Interest

The author declares that the research was conducted in the absence of any commercial or financial relationships that could be construed as a potential conflict of interest.

## Publisher's Note

All claims expressed in this article are solely those of the authors and do not necessarily represent those of their affiliated organizations, or those of the publisher, the editors and the reviewers. Any product that may be evaluated in this article, or claim that may be made by its manufacturer, is not guaranteed or endorsed by the publisher.

## Supplementary Material

Supplementary Video 1 Examples of output from Simbrain 3.0 that illustrate that a small change in a parameter can cause dramatic changes in an interconnected system.

Supplementary Video 2 This output from Simbrain 3.0 shows that when the coupling between nodes is somewhat increased (synaptic strength 0.53, set with the Synaptic adjustment dialog of Simbrain), waves of activation (i.e., infectious individuals) are easily started. However, when the coupling between nodes is somewhat decreased (synaptic strength 0.48), it is practically impossible to set off such waves (as in Supplementary Video 1).
